# Examining the link between social emotion regulation and relationship satisfaction at dyad and individual levels of analysis

**DOI:** 10.21203/rs.3.rs-9510392/v1

**Published:** 2026-05-27

**Authors:** Mayra Kalaora, Niall Bolger, Kevin N. Ochsner, Zhouzhou He

**Affiliations:** Columbia University; Columbia University; Columbia University; Columbia University

**Keywords:** Social emotion regulation, reappraisal, suppression, relationship satisfaction, romantic relationships, dyadic analysis

## Abstract

Although emotion regulation strategies such as reappraisal and suppression have been widely studied as ways individuals can self-regulate their emotions, less is known about *co*-reappraisal and *co*-suppression, and their social consequences at both the dyad and individual levels. To address these knowledge gaps, this study aimed to investigate the relationship between the two aforementioned social emotion regulation (SER) strategies — co-reappraisal and co-suppression — and relationship satisfaction in romantic couples, for both couples and the individuals within them. Using a cross-sectional survey design, 114 U.S. adult romantic couples reported on their habitual SER strategy use and relationship satisfaction. Using the Griffin and Gonzalez (1995) latent variable model, we decomposed the overall correlation between each SER strategy and relationship satisfaction into individual and dyad-level relations. We found that both couples and individuals who co-suppressed more were less satisfied with their relationship. However, co-reappraisal was not associated with relationship satisfaction at either the individual or dyad levels. These findings highlight SER as a distinct and meaningful phenomenon in the emotion regulation literature, as strategies enacted in an interpersonal, dyadic context may yield different social outcomes than what is expected from self-regulation strategies.

## Introduction

When presented with an emotional challenge, we have two options: manage difficult emotions by ourselves, or seek support from someone else. Research on emotion regulation for the past 20 years has predominantly focused on the former — the self-regulation of emotion (e.g. Gross, 2014). Yet, in everyday life, individuals frequently regulate their emotions with help from others (e.g. Coan et al., 2006; Reeck et al., 2016; Sahi et al., 2021), a process known as social emotion regulation (SER). Consistent with decades of research in the social support literature, emotion regulation researchers are increasingly examining how people seek and provide regulatory support (e.g. Dixon-Gordon, 2018; Liu et al., 2021; Tran et al., 2024; Zaki & Williams, 2013).

Despite advances in SER research, limitations remain. First, most extant work focuses on individuals as the unit of analysis (e.g. Liu et al., 2021; Tamir, 2011; Zaki & Williams, 2013). While individuals are important to study, dyads are also important to study as their own unit of analysis. In a close relationship, two individuals create a “coordinated system,” wherein tendencies emerge at the dyad level that may not be apparent at the individual level (Hudson et al., 2014). Couples can construct a joint identity (Emery, 2020) and be perceived as a joint unit (Wang et al., 2020), suggesting that dyad-level phenomena exist and warrant further attention in affective science. Second, discussion of SER strategies has rarely been integrated with their counterparts in the self-regulation literature (for exceptions, see Butler & Gross, 2009; Horn et al., 2020; Horn & Maercker, 2016). Comparing self and social versions of emotion regulation strategies can reveal if and how the latter are qualitatively different from their self-regulation counterparts. Here, we build upon canonical self-regulation strategies — reappraisal and suppression (e.g. Gross & John, 2003) — to consider the effects of *co*-reappraisal and *co*-suppression.

Third, while studies on individual outcomes, such as affect and well-being, are ubiquitous, social outcomes of social emotion regulation strategies, like relationship satisfaction, are beginning to receive more attention (English & John, 2013; Tepeli-Temiz & Elsharnouby, 2022; Lemay Jr. et al., 2024; Ruan et al., 2024). Studying social outcomes is necessary because humans are fundamentally social, and social outcomes can often be a predictor of future affective reactivity and regulation (e.g. Lin et al., 2024). Furthermore, individuals often rely on romantic partners to meet their emotion regulation needs (Haase, 2023; Heffernan et al., 2012). How these needs are met in a relationship not only influences individual outcomes but also outcomes for the dyad. Given longstanding calls to contextualize emotion regulation and its outcomes (Aldao and Plate, 2018; Doré et al., 2016), we focus our analysis on romantic couples, co-reappraisal, co-suppression, and relationship satisfaction. Specifically, this paper seeks to address the question: “How does habitual use of co-reappraisal and co-suppression relate to relationship satisfaction for both romantic couples and the individuals within them?”

## Present Research

Co-reappraisal is defined as an individual changing a situation’s meaning with another person to alter its emotional impact (Horn, 2022; Horn & Maercker, 2016). While co-reappraisal can vary within a dyad in its directionality (i.e., one person leading the reappraisal vs. joint meaning-making), the focus of co-reappraisal in this paper is an individual’s sense of a shift in perspective on a situation, achieved through talking to a close other. It is built on canonical definitions of self-reappraisal, where an individual reframes the meaning of an emotional experience in order to change its affective potency for themselves (Gross 1998, 2002; Ochsner et al. 2012). While habitual use of reappraisal is associated with better well-being overall (Aldao et al., 2010; Aldao & Nolen-Hoeksema, 2012; Webb et al., 2012), it remains unknown whether habitual use of *co*-reappraisal is beneficial, specifically for a romantic couple’s relationship satisfaction. On one hand, co-reappraisal could be positively associated with relationship satisfaction because reframing negative emotional events together can convey perceived partner responsiveness to an individual’s needs for support (Butler et al., 2003; Kardum et al., 2021; Reis et al., 2004). On the other hand, co-reappraisal could be negatively associated with relationship satisfaction: in some cases, reappraisal undermines the role of negative emotions in motivating problem-solving actions (Ford et al., 2019; Liu et al., 2023; Troy et al., 2013). Furthermore, co-reappraisal may signal to the partner that negative emotions are not acceptable (Gottman, 2011; Zhao et al., 2026), and thus reduce trust in a partner as a responsive and reliable support-provider.

Co-suppression, on the other hand, is the inhibition of one’s emotional expression due to the lack of belief in one’s partner to be an effective social regulator (Horn, 2022). Our definition of co-suppression builds upon expressive suppression in the self-regulation literature (Gross & Levenson, 1993) and in social contexts (English & John, 2013). While studies have generally found that suppression fails to provide subjective relief from negative emotions (Gross et al., 2006), the effects of co-suppression on relationship satisfaction at both the individual and dyad level are not yet well-established (Dworakowski et al., 2022). Co-suppression could lead to decreased relationship satisfaction, as co-suppression prevents the communication of emotions and responsiveness to partners’ needs, which are important for resolving conflict (Sasaki et al., 2022; Zaki et al., 2008). However, co-suppression could also benefit relationship satisfaction, given recent research suggesting that suppression could be effective when used flexibly while coping with stressful events (Aldao et al., 2015; Westphal et al., 2010).

Thus far, there are competing hypotheses about the nature of the relationship between co-reappraisal/co-suppression and relationship satisfaction. If we view *social* emotion regulation strategies as qualitatively the same as their self-regulation counterparts, then we would expect that greater habitual use of co-reappraisal is associated with greater relationship satisfaction. Similarly, greater habitual use of co-suppression would be associated with lower relationship satisfaction. If *social* emotion regulation strategies are qualitatively different from their self-regulation counterparts, then we would expect that co-reappraisal is not associated — or even negatively associated — with relationship satisfaction. Similarly, greater habitual co-suppression would not be — or even be positively associated with — relationship satisfaction.

These hypotheses can be detailed by decomposing their associations into individual and dyad-level effects. Put differently, we can ask: do couples who habitually engage in co-reappraisal report greater relationship satisfaction? We can also ask: Does the individual who engages more in co-reappraisal (relative to their partner) report more relationship satisfaction? Couple and individual-level effects do not always parallel each other (Griffin & Gonzalez, 1995), and set the stage for parsing fine-grained mechanisms of social emotion regulation strategy use. For example, if co-reappraisal use is driven by individuals, then we can expect that partners who engage more in co-reappraisal (relative to their partner) will report more relationship satisfaction. Additionally, if co-reappraisal use reflects an emergent norm between partners, then we can expect that *couples* who co-reappraise more will also report more relationship satisfaction. Similarly, if co-suppression use is driven by individuals, then we can expect that individuals who engage more in co-suppression (relative to their partner) will report less relationship satisfaction. If co-suppression use also reflects a joint process between partners, then we can also expect that couples who co-suppress more will also report less relationship satisfaction.

Taken together, we reiterate our four hypotheses. First, we hypothesized that greater co-suppression at the dyad level will be associated with less relationship satisfaction; second, at the individual level, that the partner who co-suppresses more will report lower relationship satisfaction relative to their partner. Third, that greater use of co-reappraisal at the dyad level will be associated with more relationship satisfaction; finally, that the partner who co-reappraises more will report higher relationship satisfaction relative to their partner.

## Method

This study was part of a larger dyadic study (He et al., 2026) with a daily diary component. We only report the measures and procedures related to this study in this paper.

### Recruitment and Participants

This study was approved by the Institutional Review Board (Approval Code: IRB-AAAU0758). We recruited 114 adult romantic residing in the United States who were above the age of 21 at the time of their recruitment for the study. Of those 114 couples, 97 were complete, wherein both partners’ data was available, and 17 incomplete, wherein only one partner’s data was available (n = 211). We conducted our analyses on the 97 complete dyads. The sample primarily consisted of young adult (*M*_age_ = 33.7 years, *SD*_age_= 11.8 years) heterosexual (87.6% Man-Woman, 2.1% Man-Man, 6.2% Woman-Woman and 4.1% Other) couples who have been in a relationship for at least 6 months (*M* = 7.63 years, *SD* = 8.40 years). Recruitment was done through Recruitme, Facebook, Craigslist, Reddit, Instagram, university mailing lists, and posters around New York City. We recruited 119 couples, allowing for up to 20% attrition, to obtain 80% power to detect a small to medium-sized effect in within-person estimates for the dyadic daily diary component of the study (He et al., 2026; Bolger & Laurenceau, 2013), which is not the focus of this paper. Although there is scant existing work demonstrating the effect size to be estimated for our specific research question, we estimate that we have 80% power to detect medium-sized between-person effects based on statistical guidelines and related empirical studies (Giner-Sorolla et al., 2024, Bolger & Laurenceau, 2013) for a sample of 238 participants.

### Procedure

Participants answered an individual difference survey on Qualtrics about their use of SER strategies with their romantic partner and their relationship satisfaction. Recruitment and data collection began in January 2023 and concluded by June 2023.

### Measures

#### Social Emotion Regulation Strategies

In the Interpersonal Emotion Regulation Scale for Close Relationships (IER-CR) (Horn, 2022), every item is rated on a 5-point Likert-type scale ranging from 0 (Applies not at all) to 4 (Applies fully); the results are scored by averaging the ratings for each subscale. The subscales of interest in the IER-CR were co-reappraisal and co-suppression. To illustrate these subscales, which all begin with the clause “When I am in a bad mood, or something is burdening me…,” an item measuring co-suppression is “…I act like nothing is going on because people close to me can’t help me improve my mood;” an item measuring co-reappraisal is “…I talk to a person close to me so that we can together get a new perspective of things.” Our measures co-reappraisal (α = .85) and co-suppression (α = .74) were reliable in our sample and have previously demonstrated high internal and external validity (Horn, 2022).

#### Relationship Satisfaction

The Generic Measure of Relationship Satisfaction Scale (RAS) (Hendrick, 1988) is measured on a 5-point Likert scale, ranging from 1 (low satisfaction) to 5 (high satisfaction); there are 7 items, wherein items 4 and 7 are reverse scored, and the final score is calculated through averaging the ratings. Example items are “How well does your partner meet your needs?”, or “How good is your relationship compared to most?” Our measure RAS was reliable (α = .87) and has high internal and external validity (Hendrick, 1988).

#### Data Analysis

Data was analyzed using the dplyr, ggplot2, devtools and dyadr (Garcia & Kenny, 2019) packages in R (version 4.3.3).

#### Test of Distinguishability

Distinguishability refers to whether the two members of a dyad possess a distinctive characteristic that can differentiate them in a manner that is relevant to the research question (Kenny & Ledermann, 2010; Peugh et al., 2013). Heterosexual couples are treated in the literature as de facto distinguishable dyads — and same-sex couples as indistinguishable — on the basis of gender differences (Kenny et al., 2006; Peugh et al., 2013). However, in some heterosexual dyads, gender may have no statistically significant distinguishing effect, deeming it more appropriate to treat the dyad members as indistinguishable (Kenny & Cook, 1999; Ledermann & Kenny, 2017).

We therefore wanted to empirically test the distinguishability of the data to determine the most appropriate dyad-level analysis model specification for our data. Additionally, for inclusivity purposes, we wanted to keep the non-heteronormative couples in our dataset (2.1% Man-Man, 6.2% Woman-Woman and 4.1% Other) (McGorray et al., 2023).

Using maximum likelihood estimation, we fit our data into a distinguishable model that included gender main effects, interactions, and heterogeneous variances against an indistinguishable model that constrained parameters to equality across partners using a likelihood ratio test. The test was nonsignificant for both co-reappraisal (*χ^2^*(4) = 5.11, *p* = .28) and co-suppression (*χ^2^*(4) = 4.10, *p* = .39), meaning that treating gender as a distinguishing variable did not significantly improve model fit.

We thus failed to reject the null hypothesis that the co-suppression and co-reappraisal scores on the IERCR (Horn, 2022) for women and men would have the same mean, variance, and distribution (Gonzalez & Griffin, 1997), suggesting that we can treat our entire sample as indistinguishable dyads.

#### Analysis of Individual and Dyad-Level Data

Having verified the indistinguishability of the dyads, we based our analysis on the Griffin and Gonzalez (1995) model on correlational analysis of dyad-level data, treating dyads as indistinguishable. We used the Griffin and Gonzalez (1995) model to decompose the overall correlation between SER strategies and relationship satisfaction into individual and dyad-level relations.

To address our hypotheses about dyad-level effects, we computed latent dyadic correlation r_d_, one for co-reappraisal and another for co-suppression. To address our hypotheses about individual-level effects, we computed latent individual correlation r_i_, one for co-reappraisal and another for co-suppression. We present how we calculated these latent correlations below.

The decomposition is made possible by coding each variable in a pairwise fashion so that the values for variables X and X’ (and Y and Y’) are identical except for order, where the X-columns represent each individual’s IER-CR score, and the Y-columns represent each RAS score (Table 1). Each partner is thus represented in each column, resolving the problem with indistinguishable dyads wherein there would otherwise be no meaningful way to assign one member to a specific column.

After each dyad is laid out in a pairwise manner, the Griffin & Gonzalez (1995) model computes the following correlations: overall (r_xy_), intraclass (r_xx’_ and r_yy’_), cross-intraclass (r_xy’_), individual (r_i_), and dyadic (r_d_) (Table 2).

In this paper, we computed the overall correlation r_xy_ by taking the Pearson product-moment correlation between each individual’s score on the IER-CR and their score in the RAS. The overall correlation r_xy_ answers the following question: “Are co-reappraisal/co-suppression and relationship satisfaction related?”

The two intraclass correlations r_xx’_ and r_yy’_ represent the covariance of dyad members in terms of SER and relationship satisfaction, respectively, and were also computed with the Pearson correlation. The intraclass correlations r_xx’_ and r_yy’_ answer the following question: “Do partners resemble each other on co-reappraisal, co-suppression, and relationship satisfaction?”

The cross-intraclass correlation, or r_xy’_ is the correlation between an individual’s score on the IER-CR and their partner’s score on the RAS. With r_xy’_, we sought to answer the following question: “Are dyads in which both members who co-reappraise/co-suppress more also dyads in which both members are more satisfied with their relationship?”

From these four correlations r_xy_, r_xx’_, r_yy’_, and r_xy’_, we computed latent dyadic correlation rd,and latent individual correlation r_i_. These correlations come from the assumption that the variance of an observed variable arise from two latent sources: (1) a dyadic component that represents the portion of that variable shared between the dyad (r_d_), and (2) an individual component representing the portion that is unshared and unique to the individual (r_i_) (Griffin & Gonzalez, 1995).

## Results

### Couples and Individuals who Co-Suppressed More were Less Satisfied with Their Relationship

Here, we hypothesized that higher co-suppression would be associated with lower relationship satisfaction at both the individual and the dyad level. Intraclass correlation for co-suppression (r_xx’_) was.26 and significant (*p* < .05), indicating that partners resembled each other in their use of co-suppression. As predicted, overall correlation (r_xy_ = − .38, *p* < .001) was negative and significant. Cross-intraclass correlation (r_xy’_ = − .25, *p* < .01) was also negative and significant, indicating that the dyad member who reported greater co-suppression was also likely to be the dyad member who reported lower relationship satisfaction. Latent dyadic correlation (r_d_ = − .64, *p* < .001) was also negative and significant, suggesting that couples who co-suppressed more also jointly reported lower relationship satisfaction. This suggests that the combined use of co-suppression within couples exerts a unique negative impact on their relationship satisfaction, above and beyond individual use. Furthermore, latent individual correlation (r_i_ = − .24, *p* < .05) was negative and significant. These results suggest that the partner who co-suppressed more reported lower relationship satisfaction, thus reflecting an additive between-person, within-couple effect of co-suppression on relationship satisfaction.

### Co-Reappraisal Scores in Individuals and Couples did not Predict Relationship Satisfaction

Here, we hypothesized that higher co-reappraisal within couples would be associated with greater relationship satisfaction at both the individual and dyad level. Intraclass correlation for co-reappraisal (r_xx’_) was .39 and significant (*p* < .001), indicating that partners resembled each other in their use of co-reappraisal. However, overall correlation (r_xy_= .13, *p* = .09), cross-intraclass correlation (r_xy’_ = .09, *p* = .27), individual correlation (r_i_ = .09, *p* = .36) and dyadic correlation (r_d_ = .18, *p* = .09) yielded nonsignificant results, suggesting that couples who co-reappraised more frequently were not more satisfied with their relationship than couples who co-reappraised less frequently. This suggests that the combined use of co-reappraisal within a typical couple is not associated with relationship satisfaction. Furthermore, compared to their partner, the individual who engaged more frequently in co-reappraisal did not report greater relationship satisfaction. This suggests that there are no additive, between-person effects in the use of co-reappraisal on relationship satisfaction.

## Discussion

The goal of this study was to examine the relationship between the use of social emotion regulation strategies and relationship satisfaction across couples and the individuals within them. We focused on co-reappraisal and co-suppression to determine if *social* emotion regulation strategies are qualitatively different from their widely-studied self-regulation counterparts, reappraisal and suppression (Aldao & Nolen-Hoeksema, 2012; Campbell-Sills et al., 2006; Dryman & Heimberg, 2018; Gross & John, 2003).

Additionally, we focused on relationship satisfaction given the importance of social outcomes and their interdependence with emotional outcomes (e.g. DiGiovanni & Ochsner, 2024).

To address our question, we chose the Griffin and Gonzalez (1995) model to derive, from an overall correlation, the separate dyad and individual-level effects of co-reappraisal and co-suppression on relationship satisfaction. To carry out such a decomposition would bring to light the contribution of each level of analysis to the overall correlation, and test whether dyad-level and individual-level effects could be in opposing directions (e.g., a person who co-reappraises more than their partner may be more satisfied in their relationship, but a couple that reappraises more might not be necessarily happier than other couples).

We have four main takeaways: First, couples who co-suppressed more tended to be less satisfied with their relationship. Second, individuals who co-suppressed more (relative to their partner) were also less satisfied with their relationship. Third, there was no association between how much couples co-reappraised and their relationship satisfaction. Finally, there was no association between how much individuals co-reappraised (relative to their partner) and their relationship satisfaction.

Our results on co-reappraisal suggest that *social* emotion regulation strategies might be qualitatively different from their self-regulation counterparts. These differences may be explained by the social nature of co-reappraisal — we asked participants whether they talk to *another close individual* to get a new perspective on things. When an external regulator is added to the mix, the outcomes for both the individual and the dyad become more uncertain, in that the IER-CR (Horn, 2022) captures only an *attempt* at co-reappraisal and not necessarily the eventual occurrence of reappraisal. Additionally, reappraisal could be more warranted or convincing when it comes from the self, and not so much when it comes from another. The success of a certain SER strategy is contingent on perceived support and belief in its efficacy (Williams et al, 2018). If an individual believes that SER — in our case, co-reappraisal — is unhelpful, it may lead them to construe support negatively (Bolger & Amarel, 2007; Gleason et al., 2003). With the addition of a social dimension, *co*-reappraisal may not provide the same consistent effects as self-reappraisal, which may explain why it did not predict relationship satisfaction in our sample.

We thus find novel evidence for the fit of the SER strategies of co-reappraisal and co-suppression in the context of a romantic relationship. We show that while the negative association of co-suppression and relationship satisfaction aligns with the literature that deems suppression as a self-regulation method that has negative implications for romantic relationships (Chervonsky & Hunt, 2017; Impett et al., 2012; Vater & Schröder-Abé, 2015), the positive social outcomes of reappraisal as a self-regulation strategy do not necessarily carry over once employed interpersonally in a romantic relationship, as *co*-reappraisal.

Inevitably, our study comes with limitations. First, the distribution of relationship satisfaction was significantly skewed — most couples were satisfied with their relationship (see Online Resource 1). This may affect the generalizability of our results, as couples with low relationship satisfaction in our study are still relatively satisfied couples in the population. Future research could recruit couples with low relationship satisfaction (e.g. couples in clinics). Moreover, our study adopted a cross-sectional design and cannot draw any causal claims about the directionality of the association between SER and relationship satisfaction.

This study thus examines how individuals who employ each SER strategy compare to their partner in terms of relationship satisfaction, and how couples who tend towards a certain SER strategy compare to other couples in terms of relationship satisfaction. Our findings suggest that a) adopting a dyadic approach to studying SER reveals its complexity and thus future research must study SER in the context of the individual *and* the relationship b) assessing the effectiveness of a SER strategy needs to account for the type of SER strategy and its *social* outcomes and c) co-suppression could be a promising target in further therapeutic interventions to increase relationship satisfaction.

## Supplementary Material

Supplementary Files

This is a list of supplementary files associated with this preprint. Click to download.

- OnlineResource1.docx

## Figures and Tables

**Figure 1:**
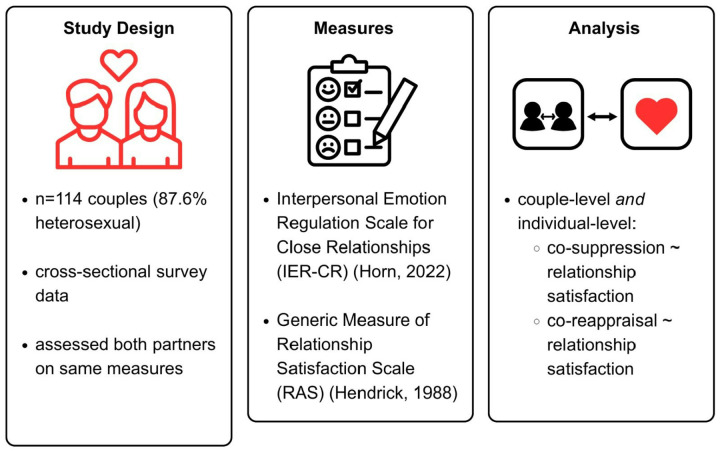
Overview of the study, with study design, measures, and analysis

**Figure 2:**
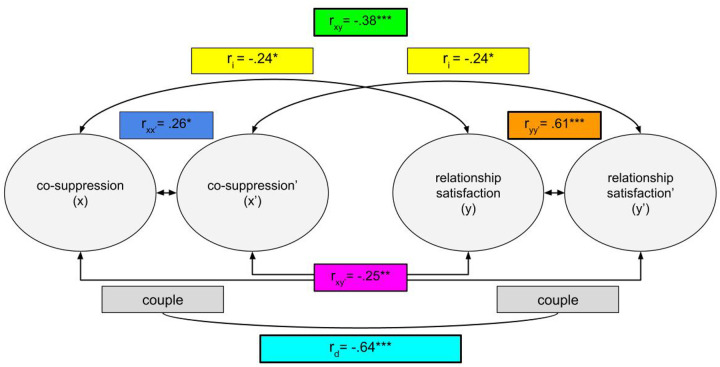
A latent variable model separating individual-level and dyad-level effects of co-suppression and relationship satisfaction. Grey circles represent unique variance for each variable, wherein both co-suppression and co-suppression’ (and relationship satisfaction and relationship satisfaction’) represent every dyad member in a pairwise fashion (see Method and Table 1). The correlations represented are overall (r_xy_), intraclass (r_xx’_ and r_yy’_), cross-intraclass (r_xy’_), individual (r_i_), and dyadic (r_d_), each assigned its own color, with “x” representing the co-suppression variable and “y” representing the relationship satisfaction variable. All coefficients shown are standardized and obtain statistical significance at the .05 level, where * is *p* < .05; ** is *p* < .01; *** is *p* < .001. Dashed arrows indicate no significant correlation, and solid arrows indicate a significant correlation; the strength of the significance, corresponding to number of asterisks, is denoted by the thickness of the box around each correlation. Created with Adobe Illustrator.

**Figure 3:**
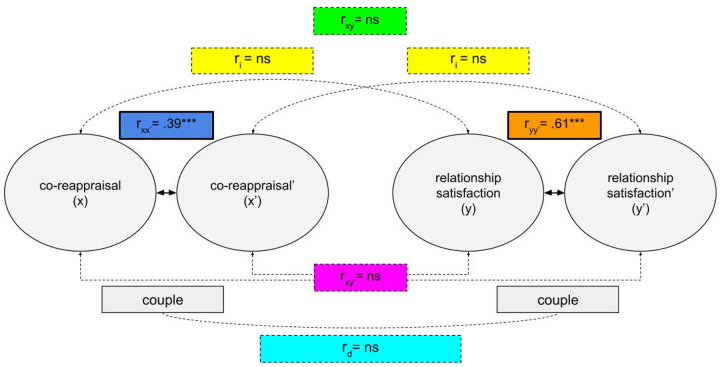
A latent variable model separating individual-level and dyad-level effects of co-reappraisal and relationship satisfaction. Grey circles represent unique variance for each variable, wherein both co-reappraisal and co-reappraisal’ (and relationship satisfaction and relationship satisfaction’) represent every dyad member in a pairwise fashion (see Method and Table 1). The correlations represented are overall (r_xy_), intraclass (r_xx’_ and r_yy’_), cross-intraclass (r_xy’_), individual (r_i_), and dyadic (r_d_), each assigned its own color, with “x” representing the co-reappraisal variable and “y” representing the relationship satisfaction variable. All coefficients shown are standardized and obtain statistical significance at the .05 level, where * is *p* < .05; ** is *p* < .01; *** is *p* < .001. Dashed arrows indicate no significant correlation, and solid arrows indicate a significant correlation; the strength of the significance, corresponding to number of asterisks, is denoted by the thickness of the box around each correlation. Dashed borders around boxes indicate no significant correlation. Created with Adobe Illustrator.

**Table 1:** Representation of the pairwise data setup for IER-CR scores and RAS scores. The first subscript represents the dyad, and the second subscript represents the individual. Categorization of individuals as 1 or 2 is arbitrary, given the indistinguishability of the dyads.

Dyad #	Variable			
	*IER-CR (X)*	*IER-CR’ (X’)*	*RAS (Y)*	*RAS’ (Y’)*
1	IER-CR_11_	IER-CR_12_	RAS_11_	RAS_12_
	IER-CR_12_	IER-CR_11_	RAS_12_	RAS_11_
2	IER-CR_21_	IER-CR_22_	RAS_21_	RAS_22_
	IER-CR_22_	IER-CR_21_	RAS_22_	RAS_21_
3	IER-CR_31_	IER-CR_32_	RAS_31_	RAS_32_
	IER-CR_32_	IER-CR_31_	RAS_32_	RAS_31_
4	IER-CR_41_	IER-CR_42_	RAS_41_	RAS_42_
	IER-CR_42_	IER-CR_41_	RAS_42_	RAS_41_

RAS = Relationship Satisfaction; IER-CR: Co-reappraisal or Co-suppression.

**Table 2:** Definitions for each type of correlation, according to Griffin and Gonzalez (1995)

Correlation	Type	Definition
r_xx’_	intraclass	how much individuals within a dyad resemble each other on a measured variable (e.g. co-suppression)
r_yy’_		
r_xy_	overall	overall Pearson correlation between each individual’s score on X and that individual’s score on Y (combines dyad- and individual-level effects)
r_xy’_	cross-intraclass	correlation between an individual′s score on variable X and their partner′s score on variable Y (only includes dyad-level effects)
r_i_	individual	individual-level effect between two variables after partialing out dyad-level effects
r_d_	dyadic	cross-intraclass correlation (r_xy’_) after correcting for unreliability in covariance across partners

## Data Availability

Data and material from the study are available through the Open Science Framework repository at: https://osf.io/hsq84/overview?view_only=7063f627b5624bfeb35ba8fa2af33843

## Code Availability

The preprocessing and analysis code is available via the Open Science Framework, also at: https://osf.io/hsq84/overview?view_only=7063f627b5624bfeb35ba8fa2af33843

## References

[R1] Aldao A., & Nolen-Hoeksema S. (2012). The influence of context on the implementation of adaptive emotion regulation strategies. Behaviour Research and Therapy, 50(7–8), 493–501. 10.1016/j.brat.2012.04.004 22659159

[R2] Aldao A., Nolen-Hoeksema S., & Schweizer S. (2010). Emotion-regulation strategies across psychopathology: A meta-analytic review. Clinical Psychology Review, 30(2), 217–237. 10.1016/j.cpr.2009.11.004 20015584

[R3] Aldao A., & Plate A. J. (2018). Coping and emotion regulation. Process-based CBT: The science and core clinical competencies of cognitive behavioral therapy (pp. 261–271). New Harbinger Publications, Inc.

[R4] Aldao A., Sheppes G., & Gross J. J. (2015). Emotion Regulation Flexibility. Cognitive Therapy and Research, 39(3), 263–278. 10.1007/s10608-014-9662-4

[R5] Bolger N., & Amarel D. (2007). Effects of social support visibility on adjustment to stress: Experimental evidence. Journal of Personality and Social Psychology, 92(3), 458–475. 10.1037/0022-3514.92.3.458 17352603

[R6] Bolger N., & Laurenceau J. P. (2013). Intensive longitudinal methods: An introduction to diary and experience sampling research (pp. xv, 256). Guilford Press.

[R7] Butler E. A., Egloff B., Wilhelm F. H., Smith N. C., Erickson E. A., & Gross J. J. (2003). The social consequences of expressive suppression. Emotion, 3(1), 48–67. 10.1037/1528-3542.3.1.48 12899316

[R8] Butler E. A., & Gross J. J. (2009). Emotion and Emotion Regulation: Integrating Individual and Social Levels of Analysis. Emotion Review, 1(1), 86–87. 10.1177/1754073908099131

[R9] Campbell-Sills L., Barlow D. H., Brown T. A., & Hofmann S. G. (2006). Acceptability and suppression of negative emotion in anxiety and mood disorders. Emotion, 6(4), 587–595. 10.1037/1528-3542.6.4.587 17144750

[R10] Chervonsky E., & Hunt C. (2017). Suppression and expression of emotion in social and interpersonal outcomes: A meta-analysis. Emotion, 17(4), 669–683. 10.1037/emo0000270 28080085

[R11] Coan J. A., Schaefer H. S., & Davidson R. J. (2006). Lending a hand: Social regulation of the neural response to threat. Psychological Science, 17(12), 1032–1039. 10.1111/j.1467-9280.2006.01832.x 17201784

[R12] DiGiovanni A. M., & Ochsner K. N. (2024). Emphasizing the Social in Social Emotion Regulation: A Call for Integration and Expansion. Affective Science, 5(3), 173–178. 10.1007/s42761-024-00260-2 39391346 PMC11461392

[R13] Dixon-Gordon K. L., Haliczer L. A., Conkey L. C., & Whalen D. J. (2018). Difficulties in interpersonal emotion regulation: Initial development and validation of a self-report measure. Journal of Psychopathology and Behavioral Assessment, 40(3), 528–549. 10.1007/s10862-018-9647-9

[R14] Doré B. P., Silvers J. A., & Ochsner K. N. (2016). Toward a Personalized Science of Emotion Regulation. Social and Personality Psychology Compass, 10(4), 171–187. 10.1111/spc3.12240 29750085 PMC5939931

[R15] Dryman M. T., & Heimberg R. G. (2018). Emotion regulation in social anxiety and depression: A systematic review of expressive suppression and cognitive reappraisal. Clinical Psychology Review, 65, 17–42. 10.1016/j.cpr.2018.07.004 30064053

[R16] Dworakowski O., Huber Z. M., Meier T., Boyd R. L., Martin M., & Horn A. B. (2022). You Do Not Have to Get through This Alone: Interpersonal Emotion Regulation and Psychosocial Resources during the COVID-19 Pandemic across Four Countries. International Journal of Environmental Research and Public Health, 19(23). 10.3390/ijerph192315699 PMC9735908 36497774

[R17] Emery L. F., Gardner W. L., Carswell K. L., & Finkel E. J. (2021). Who are We? Couple Identity Clarity and Romantic Relationship Commitment. Personality & Social Psychology Bulletin, 47(1), 146–160. 10.1177/0146167220921717 32400297

[R18] English T., & John O. P. (2013). Understanding the social effects of emotion regulation: The mediating role of authenticity for individual differences in suppression. Emotion, 13(2), 314–329. 10.1037/a0029847 23046456

[R19] Ford B. Q., Feinberg M., Lam P., Mauss I. B., & John O. P. (2019). Using reappraisal to regulate negative emotion after the 2016 U.S. Presidential election: Does emotion regulation trump political action? Journal of Personality and Social Psychology, 117(5), 998–1015. 10.1037/pspp0000200 29952576

[R20] Garcia R. L., & Kenny D. A. (2019). dyadr: Dyadic data analysis [R package]. https://github.com/RandiLGarcia/dyadr

[R21] Giner-Sorolla R., Montoya A. K., Reifman A., Carpenter T., Lewis N. A., Aberson C. L., Bostyn D. H., Conrique B. G., Ng B. W., Schoemann A. M., & Soderberg C. (2024). Power to Detect What? Considerations for Planning and Evaluating Sample Size. Personality and Social Psychology Review: An Official Journal of the Society for Personality and Social Psychology Inc, 28(3), 276–301. 10.1177/10888683241228328 38345247 PMC11193916

[R22] Gleason M. E. J., Iida M., Bolger N., & Shrout P. E. (2003). Daily supportive equity in close relationships. Personality & Social Psychology Bulletin, 29(8), 1036–1045. 10.1177/0146167203253473 15189621

[R23] Gonzalez R., & Griffin D. (1997). On the statistics of interdependence: Treating dyadic data with respect. Handbook of personal relationships: Theory, research and interventions (2nd ed., pp. 271–302). John Wiley & Sons, Inc.

[R24] Gottman J. M. (2011). The science of trust: Emotional attunement for couples (pp. xi, 480). W. W. Norton & Company.

[R25] Griffin D., & Gonzalez R. (1995). Correlational analysis of dyad-level data in the exchangeable case. Psychological Bulletin, 118(3), 430–439. 10.1037/0033-2909.118.3.430

[R26] Gross J. J. (1998). Antecedent- and response-focused emotion regulation: Divergent consequences for experience, expression, and physiology. Journal of Personality and Social Psychology, 74(1), 224–237. 10.1037//0022-3514.74.1.224 9457784

[R27] Gross J. J. (2002). Emotion regulation: Affective, cognitive, and social consequences. Psychophysiology, 39(3), 281–291. 10.1017/s0048577201393198 12212647

[R28] Gross J. J. (2014). Emotion regulation: Conceptual and empirical foundations. Handbook of emotion regulation (2nd ed., pp. 3–20). The Guilford Press.

[R29] Gross J. J., & John O. P. (2003). Individual differences in two emotion regulation processes: Implications for affect, relationships, and well-being. Journal of Personality and Social Psychology, 85(2), 348–362. 10.1037/0022-3514.85.2.348 12916575

[R30] Gross J. J., & Levenson R. W. (1993). Emotional suppression: Physiology, self-report, and expressive behavior. Journal of Personality and Social Psychology, 64(6), 970–986. 10.1037/0022-3514.64.6.970 8326473

[R31] Gross J. J., Richards J. M., & John O. P. (2006). Emotion Regulation in Everyday Life. In Emotion regulation in couples and families: Pathways to dysfunction and health (pp. 13–35). American Psychological Association. 10.1037/11468-001

[R32] Haase C. M. (2023). Emotion Regulation in Couples Across Adulthood (SSRN Scholarly Paper 4664385). Social Science Research Network. 10.1146/annurev-devpsych-120621-043836

[R33] He Z., Bolger N., & Ochsner K. (2026). What we believe about supporting others in distress: Implications for providing social regulatory support and subsequent well-being. PsyArXiv. https://osf.io/preprints/psyarxiv/qz5bu_v1

[R34] Heffernan M. E., Fraley R. C., Vicary A. M., & Brumbaugh C. C. (2012). Attachment features and functions in adult romantic relationships. Journal of Social and Personal Relationships, 29(5), 671–693. 10.1177/0265407512443435

[R35] Hendrick S. S. (1988). A generic measure of relationship satisfaction. Journal of Marriage and the Family, 50(1), 93–98. 10.2307/352430

[R36] Horn A. (2022). Interpersonal Emotion Regulation in Close Relationships Questionnaire—IER-C. 10.31234/osf.io/kmxye

[R37] Horn A. B., & Maercker A. (2016). Intra- and interpersonal emotion regulation and adjustment symptoms in couples: The role of co-brooding and co-reappraisal. BMC Psychology, 4(1), 51. 10.1186/s40359-016-0159-7 27793188 PMC5084345

[R38] Horn A. B., Zimmerli L., Maercker A., & Holzer B. M. (2023). The worse we feel, the more intensively we need to stick together: A qualitative study of couples’ emotional co-regulation of the challenge of multimorbidity. Frontiers in Psychology, 14, 1213927. 10.3389/fpsyg.2023.1213927 37637914 PMC10450955

[R39] Hudson N. W., Fraley R. C., Brumbaugh C. C., & Vicary A. M. (2014). Coregulation in Romantic Partners’ Attachment Styles: A Longitudinal Investigation. Personality & Social Psychology Bulletin, 40(7), 845–857. 10.1177/0146167214528989 24743602

[R40] Impett E. A., Kogan A., English T., John O., Oveis C., Gordon A. M., & Keltner D. (2012). Suppression sours sacrifice: Emotional and relational costs of suppressing emotions in romantic relationships. Personality & Social Psychology Bulletin, 38(6), 707–720. 10.1177/0146167212437249 22389432

[R41] Kardum I., Gračanin A., Hudek-Knežević J., & Blažić B. (2021). Emotion Regulation and Romantic Partners’ Relationship Satisfaction: Self-Reports and Partner Reports. Psihologijske Teme, 30(1), 145–159. 10.31820/pt.30.1.8

[R42] Kenny D. A., & Cook W. (1999). Partner effects in relationship research: Conceptual issues, analytic difficulties, and illustrations. Personal Relationships, 6(4), 433–448. 10.1111/j.1475-6811.1999.tb00202.x

[R43] Kenny D. A., Kashy D. A., & Cook W. L. (2006). Dyadic data analysis (pp. xix, 458). The Guilford Press.

[R44] Kenny D. A., & Ledermann T. (2010). Detecting, measuring, and testing dyadic patterns in the actor–partner interdependence model. Journal of Family Psychology, 24(3), 359–366. 10.1037/a0019651 20545409

[R45] Ledermann T., & Kenny D. A. (2017). Analyzing dyadic data with multilevel modeling versus structural equation modeling: A tale of two methods. Journal of Family Psychology, 31(4), 442–452. 10.1037/fam0000290 28165269

[R46] Lemay E. P. Jr., Teneva N., & Xiao Z. (2025). Interpersonal emotion regulation as a source of positive relationship perceptions: The role of emotion regulation dependence. Emotion, 25(2), 355–371. 10.1037/emo0001387 38900553

[R47] Lin J., Stern J. A., Allen J. P., & Coan J. A. (2024). Does attachment in adolescence predict neural responses to handholding in adulthood? A functional magnetic resonance imaging study. Journal of Social and Personal Relationships, 41(8), 2276–2296. 10.1177/02654075241239604 39166123 PMC11335342

[R48] Liu D. Y., Strube M. J., & Thompson R. J. (2021). Interpersonal Emotion Regulation: An Experience Sampling Study. Affective Science, 2(3), 273–288. 10.1007/s42761-021-00044-y 36059902 PMC9382965

[R49] Liu Z., Lu K., Hao N., & Wang Y. (2023). Cognitive Reappraisal and Expressive Suppression Evoke Distinct Neural Connections during Interpersonal Emotion Regulation. Journal of Neuroscience, 43(49), 8456–8471. 10.1523/JNEUROSCI.0954-23.2023 37852791 PMC10711701

[R50] McGorray E. L., Emery L. F., Garr-Schultz A., & Finkel E. J. (2023). Mostly White, heterosexual couples: Examining demographic diversity and reporting practices in relationship science research samples. Journal of Personality and Social Psychology, 125(2), 316–344. 10.1037/pspi0000417 36757951

[R51] Ochsner K. N., Silvers J. A., & Buhle J. T. (2012). Functional imaging studies of emotion regulation: A synthetic review and evolving model of the cognitive control of emotion. Annals of the New York Academy of Sciences, 1251, E1–24. 10.1111/j.1749-6632.2012.06751.x 23025352 PMC4133790

[R52] Peugh J. L., DiLillo D., & Panuzio J. (2013). Analyzing mixed-dyadic data using structural equation models. Structural Equation Modeling, 20(2), 314–337. 10.1080/10705511.2013.769395

[R53] Reeck C., Ames D. R., & Ochsner K. N. (2016). The Social Regulation of Emotion: An Integrative, Cross-Disciplinary Model. Trends in Cognitive Sciences, 20(1), 47–63. 10.1016/j.tics.2015.09.003 26564248 PMC5937233

[R54] Reis H. T., Clark M. S., & Holmes J. G. (2004). Perceived Partner Responsiveness as an Organizing Construct in the Study of Intimacy and Closeness. Handbook of closeness and intimacy (pp. 201–225). Lawrence Erlbaum Associates.

[R55] Ruan Y., Le J. D. V., & Reis H. T. (2024). How can I help? Specific strategies used in interpersonal emotion regulation in a relationship context. Emotion, 24(2), 329–344. 10.1037/emo0001272 37561518

[R56] Sahi R. S., Ninova E., & Silvers J. A. (2021). With a little help from my friends: Selective social potentiation of emotion regulation. Journal of Experimental Psychology General, 150(6), 1237–1249. 10.1037/xge0000853 33166161

[R57] Sasaki E., Overall N. C., Chang V. T., & Low R. S. T. (2022). A dyadic perspective of expressive suppression: Own or partner suppression weakens relationships. Emotion, 22(8), 1989–1994. 10.1037/emo0000978 34060860

[R58] Tamir M. (2011). The Maturing Field of Emotion Regulation. Emotion Review, 3(1), 3–7. 10.1177/1754073910388685

[R59] Tepeli-Temiz Z., & Elsharnouby E. (2022). Relationship Satisfaction and Well-being During the COVID-19 Pandemic: Examining the Associations with Interpersonal Emotion Regulation Strategies. Cognitive Therapy and Research, 46(5), 902–915. 10.1007/s10608-022-10317-w 35855695 PMC9275383

[R60] Tran A., Greenaway K. H., Kostopoulos J., Tamir M., Gutentag T., & Kalokerinos E. K. (2024). Does interpersonal emotion regulation effort pay off? Emotion, 24(2), 345–356. 10.1037/emo0001289 37650792

[R61] Troy A. S., Shallcross A. J., & Mauss I. B. (2013). A person-by-situation approach to emotion regulation: Cognitive reappraisal can either help or hurt, depending on the context. Psychological Science, 24(12), 2505–2514. 10.1177/0956797613496434 24145331

[R62] Vater M., & Schröder–Abé A. (2015). Explaining the Link between Personality and Relationship Satisfaction: Emotion Regulation and Interpersonal Behaviour in Conflict Discussions - Aline Vater, Michela Schröder–Abé, 2015. Sage Journals. https://journals.sagepub.com/doi/10.1002/per.1993

[R63] Wang A. M., Chen S., & Aron A. (2023). Perceiving couples as discrete units: The existence of couple-level identities. Personal Relationships, 30(3), 868–892. 10.1111/pere.12359

[R64] Webb T. L., Miles E., & Sheeran P. (2012). Dealing with feeling: A meta-analysis of the effectiveness of strategies derived from the process model of emotion regulation. Psychological Bulletin, 138(4), 775–808. 10.1037/a0027600 22582737

[R65] Westphal M., Seivert N. H., & Bonanno G. A. (2010). Expressive flexibility. Emotion, 10(1), 92–100. 10.1037/a0018420 20141306

[R66] Williams W. C., Morelli S. A., Ong D. C., & Zaki J. (2018). Interpersonal emotion regulation: Implications for affiliation, perceived support, relationships, and well-being. Journal of Personality and Social Psychology, 115(2), 224–254. 10.1037/pspi0000132 29733662

[R67] Zaki J., Bolger N., & Ochsner K. (2008). It Takes Two: The Interpersonal Nature of Empathic Accuracy. Psychological Science, 19(4), 399–404. 10.1111/j.1467-9280.2008.02099.x 18399894

[R68] Zaki J., & Williams W. C. (2013). Interpersonal emotion regulation. Emotion, 13(5), 803–810. 10.1037/a0033839 24098929

[R69] Zhao Y., Sisson N. M., Pringle V., Smith A. M., Long E. U., Elsaadawy N., Carlson E., & Ford B. Q. (2026). Managing loved ones’ emotions: The promise and pitfalls of reappraisal. Emotion. 10.1037/emo0001648 41801710

